# Comparing indoor tracking of golf ball and club metrics: Consistency and absolute agreement of the Flightscope Mevo+ and Trackman 4 launch monitors

**DOI:** 10.1016/j.jsampl.2025.100128

**Published:** 2025-12-18

**Authors:** Alex Bliss, Ben L. Langdown

**Affiliations:** a School of Education, Sport and Health Sciences, University of Brighton, Brighton, UK; b School of Education, Childhood, Youth & Sport, The Open University, Milton Keynes, UK

**Keywords:** Tracking, Technology, Club fitting, Swing analysis, Ball flight

## Abstract

**Background:**

This study aimed to compare the Flightscope Mevo ​+ ​launch monitor against a previously validated system (Trackman 4) during full golf swings in an indoor setting.

**Methods:**

Mevo+ and Trackman 4 were compared concurrently for driver (n ​= ​118, 118, 174 shots, respectively). Intraclass correlation coefficients (ICC) reported consistency and absolute agreement. Bland–Altman plots reported limits of agreement.

**Results:**

Moderate to excellent consistency was reported for all values for driver (ICC ​= ​0.66–0.996), 7-iron (ICC ​= ​0.50–0.996) and pitching wedge (ICC ​= ​0.55–1.00) except angle of attack which was poor for both 7-iron and pitching wedge (ICC ​= ​0.06 & 0.03, respectively). For absolute agreement, Mevo ​+ ​demonstrated moderate to excellent levels for most driver (ICC range ​= ​0.58–0.98), 7-iron (ICC range ​= ​0.83–0.94) and pitching wedge (ICC range ​= ​0.77–0.999) variables. Driver swing plane (ICC absolute ​= ​0.24), 7-iron angle of attack, clubhead speed, dynamic loft and spin rate (ICC absolute ​= ​0.02, 0.44, 0.23, 0.49, 0.42, respectively) and pitching wedge angle of attack, dynamic loft, and swing plane (ICC absolute ​= ​0.01, 0.25, 0.43, respectively) had poor agreement.

**Conclusion:**

Mevo+ is consistent with Trackman 4 for all variables except angle of attack. It does however provide different absolute values, but usually in a consistent, systematic manner, across a number of variables. Coaches, club fitters, golfers, and scientists should be aware of these systematic differences when attempting to compare performance across launch monitors or when solely using the Mevo ​+ ​system to aid performance, club building and fitting, or for research purposes.

## 1 Background

Utilising launch monitors to understand club and ball dynamics during the golf swing has increased in recent years [1]. The metrics provided by these devices is useful and important for golfers, golf coaches and club fitters who utilise the data to understand and interpret the golfer's swing, provide objective evidence to support technical swing changes, and make adjustments to equipment or coaching approaches. However, they are also important for sport science (biomechanics, strength and conditioning etc.) practitioners working with golfers to test and monitor their athletes and to understand the impact of any physical training programmes implemented [2].

Currently, there are two main methodologies by which launch monitors collect data: stereoscopic optical systems and Doppler radar systems. The two industry standards for these devices are the GC systems from Foresight Sports (optical) and the Trackman (Doppler radar) systems (Trackman, Denmark). Previous research has shown these systems are robust, reliable, and valid [1,3]. However, a barrier to entry for these products is cost, with the latest Trackman system at the time of writing costing ∼£20k in the United Kingdom, representing around half an average year's salary for a golf coach [4] and ∼75 ​% of the average strength and conditioning coach salary [5]. Owing to these financial constraints, a range of companies have developed increasingly affordable options that are approximately 90 ​% cheaper than the industry-leading manufacturers.

A new and affordable (∼£2000) launch monitor system released to market is the Mevo ​+ ​system from Flightscope. The Flightscope Mevo ​+ ​system combines 3D Doppler radar technology and synchronised high speed image processing to generate club and ball data [6]. However, for users to be confident in the data provided by any system, consistency and levels of agreement between industry-standard and new equipment requires investigation [7]. Recently, Brennan et al. [3], demonstrated the Mevo ​+ ​system is valid and reliable for certain metrics, but not for others, particularly around spin rates and launch or attack angles. However, their work omitted high-spinning clubs such as wedges, only investigated a limited number of ball and club parameters, and included shots where estimates of ball parameters were included. Providing assessment across clubs generating high- and low-spin rates, as well as a full range of ball and club metrics, will provide greater depth of information for practitioners using these data, such as club fitters, and high-performance golfers and golf coaches.

Therefore, the aim of this study was to compare the Mevo ​+ ​system against a previously validated, industry-standard system to determine consistency and levels of agreement during full golf swings in an indoor setting, across a large array of ball and club measures and from low- and high-spin golf clubs.

## 2 Methods

A single participant (age ​= ​36 years. Stature ​= ​1.77 ​m. Body mass ​= ​85 ​kg. Handicap Index ​= ​8.7 shots. Playing experience ​= ​18 years) performed all shots within this analysis. The location for testing was an indoor, temperature-controlled, purpose-built environment for golf swing analysis. Permission for the experiment was provided from the host organisation and the research was granted university ethical approval (∗∗∗*approval number removed for peer review∗∗∗*) and carried out in accordance with the Declaration of Helsinki (2013).

The two launch monitors used in this study were Trackman 4 (Trackman, Denmark) and Mevo+ (Flightscope HQ, 8600 Commodity Circle, Orlando, FL, USA) with Pro Package. In relation to the Trackman 4 unit, Mevo+ was positioned in front (closer to ball) and parallel, with the optical sensors aligned vertically by plumb line and confirmed with a carpenter's square and spirit level. The Mevo+ was angled as per the manufacturer instructions using the setup tool provided to ensure roll and tilt values were within normal ranges. The distance from Trackman 4 to ball was 2.5 ​m and 6 ​m to the screen. Mevo+ was 2.4 ​m to ball and 5.9 ​m to the screen. This falls within the manufacturer recommended guidelines for both for indoor usage. Alignment processes were replicated identically across three testing sessions where data were captured. Once both launch monitors were positioned as above, adhesive tape and floor markings were used to provide a repeatable standardised position between testing sessions with measurements repeated to ensure consistency. Both devices are valid for between-session usage [3] and the single-participant approach permitted a focus between unit consistency and agreement based on similar research [8].

Trackman 4 was calibrated first, prior to any data collection. Mevo+ was then calibrated, using the simulator screen to ensure target alignment from Trackman 4 was matched by Mevo ​+ ​using the FS Golf App with Pro Package (v.7.7.3), controlled via a portable tablet (Samsung Galaxy Tab A7 Lite, SM-T220). Trackman 4 was set to “normalised” which established zero (ft) altitude and wind condition with a premium ball with comparable settings on Mevo ​+ ​applied (“Standard Sea Level” and “Standard” golf ball”). The golf ball was fitted with a metallic foil circle marker housed within a dimple as per manufacturer requirements for indoor testing from Mevo+.

Following a brief warm up, the participant completed full shots with a driver (Callaway XR Pro 16, 10°), 7-iron and pitching wedge (both Titleist 718 AP2) in a random order using premium golf balls (Titleist Pro V1x). Drive shots were hit from a “castle” tee, allowing for consistent tee height (40 ​mm). Iron shots were hit directly from an artificial turf mat.

### 2.1 Statistical analysis

Data were exported from Trackman 4 and FS Golf App/cloud platform and organised in Microsoft Excel. The following number of shots were performed with each club: Driver n ​= ​118.7-iron n ​= ​118. Pitching wedge n ​= ​174. To be included for analysis, all ball and club variables were required to be collected for each shot performed. A three-part exclusion process was employed:

1) Any missing data from a shot resulted in the removal of all data for that shot from the analysis across both launch monitors.
2) Spin rate for both monitors are either “measured” or “estimated”. Where spin rate was estimated, the shot was removed from the analysis across both launch monitors.
3) As severely mishit shots (shanks, tops etc.) would not be used in coaching or club fitting, these shots were excluded based on the following pre-defined threshold values: Driver carry distance= <150 yards, 7iron carry distance= <100 yards. PW carry distance = <50 yards.

The remaining data (driver n ​= ​58. 7-iron n ​= ​80. PW n ​= ​69) were analysed in Statistical Package for the Social Sciences (SPSS, v26, IBM). Reasons for data removal were: Driver: n ​= ​3 shots below 150 yards carry; n ​= ​15 no club data captured on Trackman 4; n ​= ​42 removal due to either Trackman 4 or Mevo ​+ ​reporting estimated spin rates. 7-iron: n ​= ​7 no club data captured on Trackman 4; n ​= ​31 estimated spin rates on Mevo+. PW: n ​= ​60 no club data captured on Trackman 4; n ​= ​4 no club data from Mevo+; n ​= ​41 estimated spin rates from Mevo+. An *a priori two-tailed* power analysis was conducted in G∗Power, determining that based on an alpha level of 0.05 with an anticipated correlation of 0.5, that 29 shots were required to achieve beta power of 0.8. Data were assessed for normality using interpretation of skewness and kurtosis values as well as visual inspection of histograms and Q–Q plots. Effect sizes were calculated using Cohen's *d* and interpreted with the following thresholds: <0.2 *trivial*; 0.2–0.5 *small*; 0.5–0.8 ​= ​*moderate*; >0.8 *large* [9].

Intraclass correlation coefficients (ICC) and their 95 ​% confidence intervals were calculated using a mean-rating, two-way mixed-effects alpha model with consistency and absolute agreement reported. ICC consistency refers to the correlation between two measures in an additive manner and absolute agreement reports to what extent the same score is achieved between samples [10]. Simply, consistency equates to *y ​= ​x ​+ ​c* where *y* and *x* are the two samples collected and *c* is the systematic error. Absolute agreement concerns the extent to which *y ​= ​x* [10]. Results from the ICC analyses were characterised as: <0.5 ​= ​“poor”, 0.5–0.75 ​= ​“moderate”, 0.75–0.9 ​= ​“good”, >0.9 ​= ​excellent [10]. Bland–Altman plots were created to represent the agreement between the two systems, with upper and lower bounds establish as 1.96∗SD above and below the mean, respectively. It is recommended with Bland–Altman analysis that acceptable agreement limits are established *a priori* based on multiple factors, including clinical relevance and practitioner experience [11]. Therefore, *a priori* agreement was set at 95 ​% of data points falling within the 95 ​% confidence limits for each metric compared across devices, or practically, one shot within a 20-shot session demonstrating agreement outside of the 95 ​% confidence limits.

## 3 Results

All data are presented in the Tables and Figures herein.

**Fig. 1:**
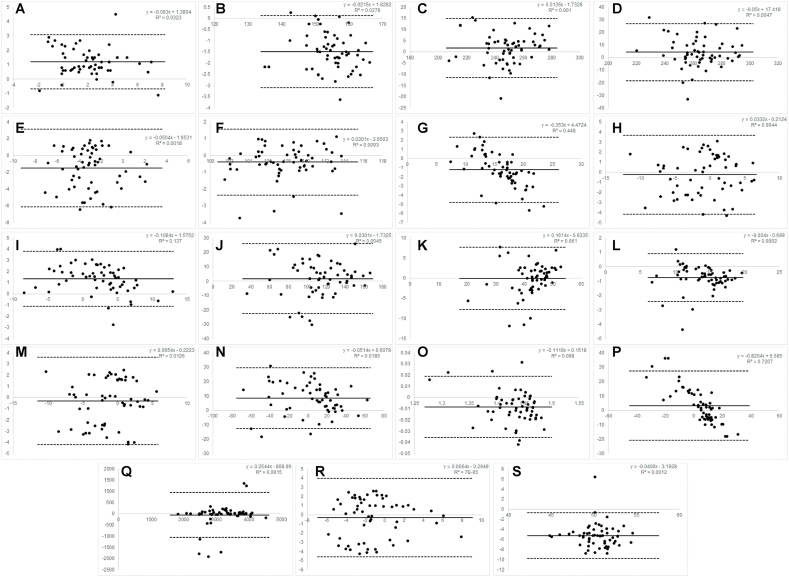
Bland–Altman plots for Driver.

**Fig. 2:**
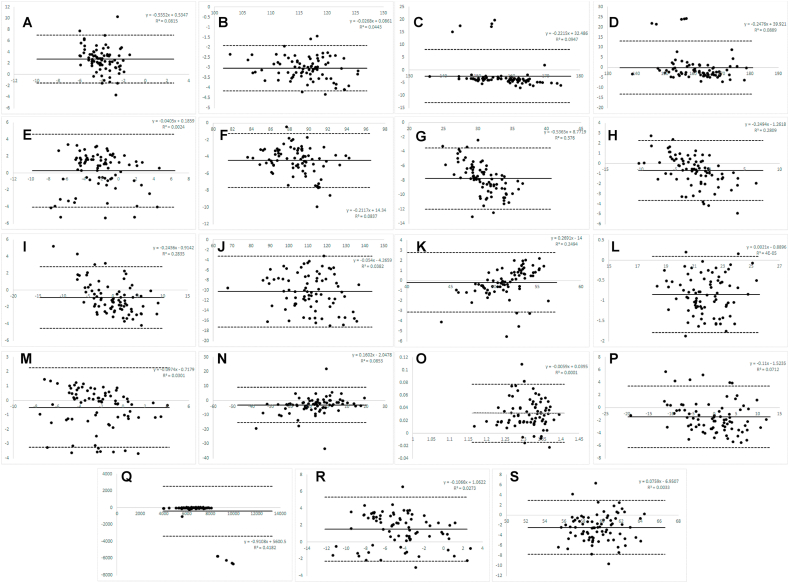
Bland–Altman plots for 7-iron.

**Fig. 3:**
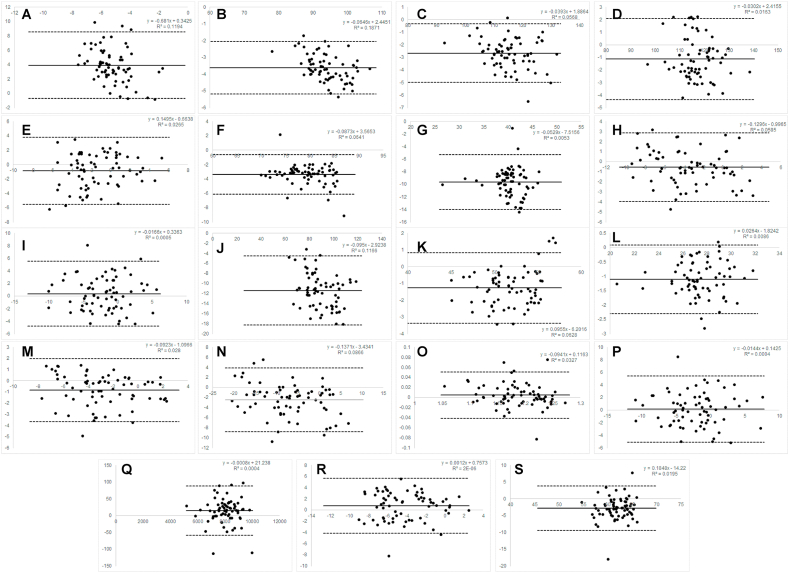
Bland–Altman plots for pitching wedge.

Fig. 1, Fig. 2, Fig. 3 note: A ​= ​Angle of Attack; B= Ball Speed; C= Carry Distance; D ​= ​Total Distance; E ​= ​Club Path; F=CHS; G ​= ​Dynamic Loft; H= Face Angle; I= Face to Path; J ​= ​Height; K= Landing Angle; L ​= ​Launch Angle; M ​= ​Launch Direction; N ​= ​Dispersion (Side); O= Smash Factor; P= Spin Axis; Q ​= ​Spin Rate; R= Swing Direction; S= Swing Plane. Y axis values are the mean difference. X axis values are the unit of measure for the variable. These correspond with the units of measure contained in Table 1, Table 2, Table 3.

**Table 1:** Descriptive statistics and correlations for driver.

N ​= ​58	Trackman		Mevo+		Effect Size (d)	Mean Difference	MAPE (%)	ICC (Consistency) (95 ​% CI)	ICC Descriptor	ICC (Absolute) (95 ​% CI)	ICC Descriptor	r ​=
Variable	Mean ​± ​SD	Range (min–max)	Mean ​± ​SD	Range (min–max)								
Angle of attack (°)	2.73 ​± ​2.05	−2.30–7.70	1.53 ​± ​2.21	−2.60–8.20	0.56	1.20 ​± ​0.96	43.97	0.95 (0.91, 0.97)	E	0.87 (0.09, 0.96)	PE	0.90
Ball speed (mph)	153.82 ​± ​6.28	133.84–165.14	155.31 ​± ​6.42	136.00–167.10	0.23	−1.49 ​± ​0.82	0.97	0.996 (0.993, 0.998)	E	0.98 (0.36, 0.996)	PE	0.99
Carry distance (yds)	249.37 ​± ​16.40	205.54–283.93	247.73 ​± ​16.19	209.40–277.40	0.10	1.63 ​± ​6.72	0.65	0.96 (0.93, 0.97)	E	0.95 (0.92, 0.97)	E	0.92
Total distance (yds)	266.23 ​± ​16.45	222.69–302.56	262.02 ​± ​17.19	212.80–291.90	0.25	4.21 ​± ​11.53	1.58	0.87 (0.78, 0.92)	G-E	0.85 (0.74, 0.92)	M-E	0.77
Club path (L/R)	−3.75 ​± ​2.28	−9.30–1.70	−2.25 ​± ​2.37	−6.90–3.60	0.65	−1.50 ​± ​2.35	40.00	0.66 (0.42, 0.80)	P-G	0.58 (0.19, 0.77)	P-G	0.49
Club speed (mph)	107.70 ​± ​3.29	100.77–113.72	108.10 ​± ​3.19	102.10–115.40	0.12	−0.40 ​± ​1.00	0.37	0.98 (0.96, 0.99)	E	0.97 (0.95, 0.99)	E	0.95
Dynamic loft (°)	15.55 ​± ​2.91	7.28–20.96	16.78 ​± ​4.11	7.60–26.10	0.35	−1.23 ​± ​1.82	7.91	0.93 (0.88, 0.96)	E	0.90 (0.71, 0.96)	M-E	0.92
Face angle (°)	−1.01 ​± ​4.17	−11.10–6.63	−0.76 ​± ​4.04	−12.40–6.70	0.06	−0.24 ​± ​2.00	23.76	0.94 (0.89, 0.96)	E	0.94 (0.90, 0.96)	E	0.88
Face to path (L/R)	2.83 ​± ​4.07	−8.38–13.02	1.49 ​± ​4.53	−8.50–12.80	0.31	1.34 ​± ​1.25	47.35	0.98 (0.96, 0.89)	E	0.96 (0.62, 0.99)	M-E	0.96
Height (ft)	114.30 ​± ​28.42	31.76–169.56	112.62 ​± ​27.61	40.20–163.80	0.06	1.68 ​± ​12.33	1.47	0.95 (0.91, 0.97)	E	0.95 (0.91, 0.97)	E	0.90
Land angle (°)	42.80 ​± ​6.78	17.49–54.51	42.83 ​± ​5.85	23.10–51.40	0.01	−0.02 ​± ​3.93	0.05	0.89 (0.82, 0.94)	G-E	0.90 (0.82, 0.94)	G-E	0.81
Launch angle (°)	13.61 ​± ​2.92	6.14–19.36	14.36 ​± ​2.93	7.20–19.80	0.26	−0.75 ​± ​0.84	5.51	0.98 (0.96, 0.99)	E	0.96 (0.79, 0.99)	G-E	0.96
Launch Dir. (L/R)	−1.40 ​± ​3.66	−10.02–5.84	−1.10 ​± ​3.45	−11.50–5.30	0.08	−0.30 ​± ​1.99	21.43	0.92 (0.86, 0.95)	E	0.92 (0.86, 0.95)	G-E	0.85
Side (L/R)	7.28 ​± ​28.24	−66.60–65.70	−1.17 ​± ​29.68	−75.20–58.50	0.29	8.45 ​± ​10.74	116.07	0.96 (0.94, 0.98)	E	0.94 (0.77, 0.98)	G-E	0.93
Smash factor (AU)	1.43 ​± ​00.04	1.29–1.47	1.44 ​± ​0.04	1.27–1.50	0.25	−0.01 ​± ​0.01	0.70	0.97 (0.95, 0.98)	E	0.96 (0.89, 0.98)	G-E	0.94
Spin axis (L/R)	5.82 ​± ​8.17	−21.86–22.74	2.58 ​± ​18.21	−45.00–39.30	0.23	3.24 ​± ​12.28	55.67	0.77 (0.61, 0.86)	M-G	0.76 (0.59, 0.86)	M-G	0.83
Spin rate (rPm)	3171 ​± ​694	1581–4551	3226 ​± ​560	1825–4610	0.09	−55 ​± ​513	1.73	0.80 (0.66, 0.88)	M-G	0.80 (0.67, 0.88)	M-G	0.68
Swing Dir. (L/R)	−1.34 ​± ​3.06	−6.92–6.71	−1.04 ​± ​3.04	−5.70–9.10	0.10	−0.30 ​± ​2.18	22.39	0.85 (0.75, 0.91)	G-E	0.85 (0.75, 0.91)	G-E	0.74
Swing plane (°)	47.77 ​± ​2.25	42.25–53.35	53.01 ​± ​2.32	46.90–57.60	2.29	−5.24 ​± ​2.31	10.97	0.66 (0.42, 0.80)	P-G	0.24 (−0.14, 0.58)	P-M	0.49

MAPE ​= ​Mean Average Percentage Difference. E ​= ​Excellent. G ​= ​Good. M ​= ​Moderate. P= Poor.

**Table 2:** Descriptive statistics and correlations for 7 Iron.

N ​= ​80	Trackman		Mevo+		Effect Size (d)	Mean Difference	MAPE (%)	ICC (Consistency) (95 ​% CI)	ICC Descriptor	ICC (Absolute) (95 ​% CI)	**ICC Descriptor**	**r=**
Variable	Mean ​± ​SD	Range (min–max)	Mean ​± ​SD	Range (min–max)								
Angle of attack (°)	−2.58 ​± ​1.31	−5.10–2.60	−5.31 ​± ​1.76	−9.90–0.90	1.76	2.73 ​± ​2.16	105.81	0.06 (−0.47, 0.39)	P	0.02 (−0.14, 0.20)	P	0.29
Ball speed (mph)	114.69 ​± ​4.46	101.56–123.85	117.72 ​± ​4.58	103.90–127.00	0.67	−3.03 ​± ​0.57	2.64	0.996 (0.994, 0.997)	E	0.90 (−0.02, 0.97)	P-E	0.99
Carry distance (yds)	156.34 ​± ​7.09	134.68–171.38	158.75 ​± ​8.65	135.00–177.40	0.30	−2.41 ​± ​5.35	1.54	0.87 (0.80, 0.92)	G-E	0.85 (0.72, 0.91)	M-E	0.79
Total distance (yds)	161.41 ​± ​7.76	137.09–178.56	161.47 ​± ​9.61	134.40–181.00	0.01	−0.06 ​± ​6.70	0.04	0.83 (0.73, 0.89)	M-G	0.83 (0.73, 0.89)	M-G	0.72
Club path (L/R)	−2.06 ​± ​2.85	−9.80–4.90	−2.34 ​± ​2.96	−8.20–6.40	0.10	0.28 ​± ​2.21	13.59	0.83 (0.74, 0.89)	M-G	0.83 (0.74, 0.89)	M-G	0.71
Club speed (mph)	86.49 ​± ​2.15	81.57–92.15	90.93 ​± ​2.59	85.60–96.50	1.87	−4.44 ​± ​1.64	5.13	0.87 (0.79, 0.91)	G-E	0.44 (−0.12, 0.78)	P-G	0.78
Dynamic loft (°)	26.94 ​± ​2.00	22.37–32.36	34.71 ​± ​3.24	26.50–40.70	2.89	−7.77 ​± ​2.16	28.84	0.81 (0.70, 0.88)	M-G	0.23 (−0.07, 0.59)	P-M	0.76
Face angle (°)	−2.65 ​± ​2.89	−10.16–5.65	−1.97 ​± ​3.67	−10.20–7.60	0.21	−0.69 ​± ​1.51	26.04	0.95 (0.91, 0.96)	E	0.94 (0.88, 0.96)	G-E	0.92
Face to path (L/R)	−1.31 ​± ​3.22	−10.16–7.40	0.37 ​± ​4.64	−14.60–10.20	0.42	−0.90 ​± ​1.87	68.70	0.87 (0.79, 0.92)	G-E	0.83 (0.60, 0.91)	M-E	0.82
Height (ft)	105.68 ​± ​12.70	62.75–132.31	115.93 ​± ​13.39	72.20–143.70	0.79	−10.25 ​± ​3.57	9.70	0.98 (0.97, 0.99)	E	0.85 (−0.09, 0.96)	P-E	0.96
Land angle (°)	51.28 ​± ​3.23	38.24–56.44	51.46 ​± ​2.50	41.60–57.20	0.06	−0.18 ​± ​1.50	3.51	0.93 (0.89, 0.95)	G-E	0.93 (0.89, 0.95)	G-E	0.89
Launch angle (°)	21.37 ​± ​1.47	18.07–25.11	22.21 ​± ​1.47	18.70–25.60	0.57	−0.84 ​± ​0.48	3.93	0.97 (0.96, 0.98)	E	0.90 (−0.08, 0.97)	P-E	0.95
Launch Dir. (L/R)	−2.59 ​± ​2.47	−8.53–3.88	−2.10 ​± ​2.70	−7.90–4.20	0.19	−0.49 ​± ​1.40	18.91	0.92 (0.88, 0.95)	G-E	0.91 (0.85, 0.95)	G-E	0.86
Side (L/R)	−8.64 ​± ​12.66	−47.25–20.10	−5.46 ​± ​10.89	−30.70–18.90	0.27	−3.18 ​± ​6.25	36.81	0.93 (0.88, 0.95)	G-E	0.91 (0.81, 0.95)	G-E	0.87
Smash factor (AU)	1.33 ​± ​0.05	1.17–1.41	1.29 ​± ​0.05	1.16–1.38	0.80	0.03 ​± ​0.02	2.26	0.93 (0.90, 0.96)	E	0.83 (−0.06, 0.95)	P-E	0.87
Spin axis (L/R)	−1.17 ​± ​5.82	−19.79–10.94	0.30 ​± ​6.47	−18.60–12.80	0.24	−1.48 ​± ​2.48	126.50	0.96 (0.93, 0.97)	E	0.94 (0.86, 0.97)	G-E	0.92
Spin rate (rpm)	6390 ​± ​821	3967–8079	6795 ​± ​1665	4013–13289	0.31	−405 ​± ​1512	6.33	0.50 (0.23, 0.68)	P-M	0.49 (0.21, 0.67)	P-M	0.43
Swing Dir. (L/R)	−3.79 ​± ​3.01	−12.16–2.39	−5.34 ​± ​3.31	−11.30–3.50	0.49	1.55 ​± ​1.94	40.90	0.90 (0.84, 0.93)	G-E	0.84 (0.48, 0.93)	P-E	0.82
Swing plane (°)	58.36 ​± ​2.52	52.26–64.55	60.78 ​± ​2.39	54.80–66.60	0.99	−2.43 ​± ​2.71	4.16	0.56 (0.32, 0.72)	P-M	0.42, (−0.07, 0.67)	P-M	0.39

MAPE ​= ​Mean Average Percentage Difference. E ​= ​Excellent. G ​= ​Good. M ​= ​Moderate. P= Poor.

**Table 3:** Descriptive statistics and correlations for Pitching Wedge.

N ​= ​69	Trackman		Mevo+		Effect Size (d)	Mean Difference	MAPE (%)	ICC (Consistency) (95 ​% CI)	ICC Descriptor	ICC (Absolute) (95 ​% CI)	ICC Descriptor	r ​=
Variable	Mean ​± ​SD	Range (min–max)	Mean ​± ​SD	Range (min–max)								
Angle of attack (°)	−3.29 ​± ​1.17	−6.70–−0.20	−7.18 ​± ​2.07	−11.0–−1.90	2.31	3.91 ​± ​2.36	118.84	0.03 (−0.57, 0.40)	P	0.01 (−0.11, 0.15)	P	0.02
Ball speed (mph)	92.10 ​± ​5.26	76.80–104.62	95.75 ​± ​5.44	79.30–108.30	0.68	−3.62 ​± ​0.80	3.93	0.995 (0.992, 0.997)	E	0.89 (−0.03, 0.98)	P-E	0.99
Carry distance (yds)	114.17 ​± ​7.17	91.50–130.47	116.83 ​± ​7.31	94.00–133.30	0.37	−2.66 ​± ​1.19	2.33	0.993 (0.99, 0.996)	E	0.96 (0.02, 0.99)	P-E	0.99
Total distance (yds)	116.61 ​± ​6.95	96.17–136.48	117.80 ​± ​7.00	97.40–140.40	0.17	−1.13 ​± ​1.64	0.97	0.99 (0.98, 0.992)	E	0.98 (0.92, 0.992)	E	0.97
Club path (L/R)	−1.77 ​± ​2.85	−9.00–4.50	−0.93 ​± ​2.84	−6.20–6.10	0.08	−0.87 ​± ​2.37	49.15	0.78 (0.65, 0.87)	M-G	0.77 (0.61, 0.86)	M-G	0.65
Club speed (mph)	77.64 ​± ​3.95	68.48–84.74	81.00 ​± ​4.30	71.30–89.20	0.81	−3.36 ​± ​1.40	4.33	0.97 (0.95, 0.98)	E	0.83 (−0.12, 0.96)	P-E	0.95
Dynamic loft (°)	35.57 ​± ​2.61	25.97–41.91	45.25 ​± ​3.77	27.00–51.00	2.99	−9.66 ​± ​2.22	27.16	0.87 (0.79, 0.92)	G-E	0.25 (−0.05, 0.62)	P-M	0.82
Face angle (°)	−3.72 ​± ​2.92	−9.76–1.47	−3.15 ​± ​3.75	−10.60–4.80	0.17	−0.55 ​± ​1.74	14.79	0.93 (0.88, 0.96)	G-E	0.92 (0.87, 0.95)	G-E	0.89
Face to path (L/R)	−1.89 ​± ​3.17	−8.24–5.51	−2.22 ​± ​4.18	−13.00–6.20	0.09	0.37 ​± ​2.64	19.58	0.85 (0.76, 0.91)	G-E	0.85 (0.76, 0.91)	G-E	0.77
Height (ft)	83.30 ​± ​12.05	60.85–113.65	94.77 ​± ​13.38	65.20–123.20	0.90	−11.38 ​± ​3.51	13.66	0.98 (0.97, 0.99)	E	0.81 (−0.07, 0.96)	P-E	0.97
Land angle (°)	51.14 ​± ​3.20	43.96–57.75	52.39 ​± ​2.50	46.00–56.30	0.44	−1.26 ​± ​1.07	2.46	0.96 (0.94, 0.98)	E	0.92 (0.34, 0.97)	P-E	0.96
Launch angle (°)	26.80 ​± ​2.16	18.53–31.41	27.90 ​± ​2.17	19.40–32.30	0.51	−1.10 ​± ​0.61	4.10	0.98 (0.97, 0.99)	E	0.92 (−0.05, 0.98)	P-E	0.96
Launch Dir. (L/R)	−3.25 ​± ​2.54	−8.66–1.77	−2.41 ​± ​2.86	−7.80–3.30	0.31	−0.84 ​± ​1.44	25.84	0.93 (0.88, 0.95)	G-E	0.90 (0.76, 0.95)	G-E	0.87
Side (L/R)	−8.40 ​± ​6.96	−21.96–3.89	−5.89 ​± ​7.35	−20.00–10.30	0.35	−2.45 ​± ​3.25	29.17	0.95 (0.91, 0.97)	E	0.92 (0.69, 0.97)	Mod-E	0.90
Smash factor (AU)	1.19 ​± ​0.05	1.05–1.26	1.18 ​± ​0.05	1.06–1.29	0.2	0.00 ​± ​0.02	0.00	0.93 (0.89, 0.96)	G-E	0.93 (0.89, 0.96)	G-E	0.88
Spin axis (L/R)	−2.11 ​± ​3.68	−10.11–7.80	−2.24 ​± ​4.46	−12.00–7.70	0.03	0.17 ​± ​2.68	8.06	0.88 (0.80, 0.92)	G-E	0.88 (0.80, 0.92)	G-E	0.79
Spin rate (rpm)	8031 ​± ​886	5186–9909	8016 ​± ​891	5193–10021	0.02	14 ​± ​37	0.17	1.00 (0.999, 1.00)	E	0.999 (0.999, 1.00)	E	1.00
Swing Dir.(L/R)	−3.79 ​± ​3.06	−10.82–2.49	−4.56 ​± ​2.91	−10.10–2.50	0.26	0.75 ​± ​2.50	0.20	0.78 (0.65, 0.86)	M-G	0.77 (0.62, 0.86)	M-G	0.64
Swing plane (°)	60.42 ​± ​3.44	45.61–69.86	63.15 ​± ​2.68	−57.00–68.20	0.89	−2.80 ​± ​3.38	0.05	0.55 (0.27, 0.72)	P-M	0.43 (−0.02, 0.67)	P-M	0.39

MAPE ​= ​Mean Average Percentage Difference. E ​= ​Excellent. G ​= ​Good. M ​= ​Moderate. P= Poor.

*Driver:* Mevo ​+ ​demonstrated moderate to excellent consistency (ICC range ​= ​0.66–0.996) with Trackman 4 across all 19 recorded variables. It demonstrated moderate to excellent absolute agreement (ICC range ​= ​0.58–0.98) for all variables, except swing plane which was poor (ICC absolute ​= ​0.24).

*7-iron:* Mevo ​+ ​demonstrated moderate to excellent consistency (ICC range ​= ​0.50–0.996) with Trackman 4 for all variables, except angle of attack which was poor (ICC consistency ​= ​0.06). It demonstrated good to excellent absolute agreement (ICC range ​= ​0.83–0.94) for all variables except angle of attack, clubhead speed, dynamic loft, spin rate and swing plane which were all poor (ICC absolute ​= ​0.02, 0.44, 0.23, 0.49, 0.42, respectively).

*Pitching Wedge:* Mevo ​+ ​demonstrated moderate to excellent consistency (ICC range ​= ​0.55–1.00) with Trackman 4 for all variables, except angle of attack which was poor (ICC consistency ​= ​0.03). It demonstrated good to excellent absolute agreement across all variables (ICC range ​= ​0.77–0.999) except angle of attack, dynamic loft, and swing plane which were all poor (ICC absolute ​= ​0.01, 0.25, 0.43, respectively).

Confidence intervals for ICC consistency measures were generally robust and had close proximity to the ICC value, however a number of measures had wide confidence intervals (crossing the “0” threshold), particularly for absolute agreement (see Table 1, Table 2, Table 3).

## 4 Discussion

The aim of this study was to compare the Mevo ​+ ​system against a previously validated system (Trackman 4) to determine consistency and agreement during full golf swings within an indoor practice setting. The main findings were that, across 19 variables and three club types investigated, Mevo ​+ ​demonstrated moderate to excellent levels of consistency with Trackman 4 for all measures except angle of attack with pitching wedge and 7-iron. Results demonstrated moderate to excellent absolute agreement across all variables, with the exception of angle of attack and dynamic loft and with 7-iron and pitching wedge, club speed and spin rate for 7-iron, and swing plane for all three clubs. This suggests Mevo ​+ ​demonstrates acceptable levels of consistency when compared to the Trackman 4 for all variables except angle of attack. However, Mevo ​+ ​provides different absolute values, but in a consistent manner across a number of variables. Users should be aware of these differences if comparing across launch monitors or when solely using the Mevo ​+ ​system to aid performance, fit clubs, or for research purposes as direct comparison will provide different actual values, but in a consistent manner.

Establishing levels of agreement between golf launch monitors is important, particularly for golfers, golf coaches, and club fitters, especially if multiple systems are being utilised. This may occur in applied, research, and teaching settings where practitioners are attempting to compare data across units (e.g., where a golfer practices or has coaching sessions at different facilities). Additionally, understanding levels of agreement between launch monitors will support practitioners who are considering an alternative device to their current system or utilising objective data to inform a purchase decision for a particular system.

While the Trackman 4 has been shown to be reliable and valid previously [1], to date, only two studies have investigated test-retest reliability [12] or reliability and validity of the Mevo ​+ ​[3], however, neither utilised low- and high-spinning clubs across a full range of swing metrics, as has been conducted herein. This is necessary to allow for comprehensive analysis of the performance of the Mevo ​+ ​system and for users to have better understanding of the system's consistency and absolute agreement with the criterion Doppler radar system, Trackman 4.

Brennan et al. [3] established Mevo ​+ ​has acceptable reliability and validity when monitoring CHS, ball speed, carry distance and smash factor. However, spin rate, launch angle and attack angle were different between Mevo+ and Trackman 4. The work of Brennan et al. [3] utilised a noticeably different methodological approach to the work presented in this study, which may explain the differences in findings. For example, where spin rate ICCs were reported as “poor” in Brennan et al. [3], in this study, for driver and pitching wedge ICCs were “moderate to good” or “excellent”, respectively, with pitching wedge obtaining an ICC statistic of 1.00 for pitching wedge. The contrasting findings can be explained by the approach used here of eliminating shots where the launch monitors could only “estimate” spin rates. By only including “measured” shots across both units per shot in this analysis, errors associated with the monitor “estimating” spin rates was eliminated. An estimate, by its nature, is likely to be inaccurate when compared to a measurement. By eliminating estimated shot variables, a potential source of error was removed. All shots, including those with estimated spin rates were included in the analysis in Brennan et al. work [3]. For example, some driver shots in Brennan et al. had spin rates of ∼12,000 ​rpm. This is highly unlikely to be a measured spin rate, even with a poor strike location and club delivery profile. Therefore, either the Trackman 4 or Mevo ​+ ​would have provided an estimated spin rate. For example, Leach et al. [1] demonstrated spin rates for driver in recreational and skilled golfers were typically between ∼2000–5000 ​rpm. It is recommended therefore, as would happen in applied practice if undertaking a professional club fitting or coaching lesson, that to improve comparability between launch monitors and improve accuracy that only measured shots are used.

The above notwithstanding, it should be noted that for 7-iron spin rate data in the present study also found the consistency between launch monitors to be “poor-moderate” (ICC consistency ​= ​0.50). This decrease for 7-iron versus other clubs in the study is likely a result of certain shots being reported as “measured” when the data obtained demonstrate that this is unlikely. The Mevo ​+ ​units on five occasions across the 80 shots analysed reported extremely high spin rates. In a study involving golfers of similar playing ability to the participant in this study, spin rate with 8-iron was 5491 ​± ​1523 ​rpm [13]. The spin rates on the five occasions mentioned in this study were between 11,000 and 13,500 ​rpm, around 6000 ​rpm higher than was reported on the Trackman 4. This error occurs when the Mevo ​+ ​unit estimates the spin rate, but for each example included here, spin rates were recorded as “measured” and therefore did not violate the exclusion criteria and were included in the analysis. It is likely that in these instances the doppler radar used to detect the spin rate has “doubled” the spin as a result of a seam error, where the seam of the golf ball modulates the doppler signal at twice the rate as has been reported elsewhere [14]. These errors only occurred in small number of shots (5/80 or 6 ​%) and were isolated to 7-iron. Although included here, in applied settings, a coach, player, or club fitter would quickly disregard a shot that was struck well, and had normal flight and distance, but exhibited a “measured” 13,000 ​rpm spin rate as this is extremely unlikely to occur. Therefore, while the Mevo+ is consistent and accurate for the majority of metrics when compared to Trackman 4, users of Mevo ​+ ​should be vigilant data screening eliminate rogue data from their sessions.

Angle of attack showed poor consistency across 7-iron and pitching wedge. Angle of attack is the movement of the golf club head at impact when projected onto a vertical plane [13]. Angle of attack, together with clubface loft presented at impact determine dynamic loft [13]. Launch angle is influenced by dynamic loft and the impact location [15]. Similarly, spin rates are influenced by clubhead speed, strike location, and angle of attack/dynamic loft [15]. Therefore, practitioners should exercise caution when utilising Mevo ​+ ​when comparing data against a Trackman 4 for angle of attack measures.

The approach to establish ICCs within this study were to use consistency and absolute agreement. This dual ICC reporting approach is considered best practice [10]. While ICC consistency measures were generally robust here, demonstrating high ICC values and small confidence intervals around the ICC value, a number of ICC absolute measures had wide confidence intervals (crossing the “0” threshold). For example, ball speed for 7-iron had an ICC absolute value of 0.90, but a confidence interval from −0.02 to 0.97. It did however demonstrate almost perfect consistency (ICC ​= ​0.996). Consequently, practitioners and golfers should note that while the Mevo ​+ ​might demonstrate good or excellent consistency with Trackman 4, it may have poor absolute agreement in the same metric. Therefore, ICC absolute values reported should be carefully considered on an individual variable basis.

To control extraneous variables, strict inclusion criteria utilising only trials where both systems captured all ball and club data, meaning severe mishits and any shots with estimated spin rates were removed. This resulted in a relatively high number of trials being discounted for some clubs, notably pitching wedge and driver. This robust approach allowed for direct comparison of measurements, rather than the inherent variability associated with the launch monitors estimating parameters. Similar issues with collecting launch monitor club data in an indoor setting have been reported previously [1]. A limitation herein is that, while the Trackman 4 unit is validated for research purposes and widely adopted in the industry, it is not technically a “gold-standard” (largely considered to be motion capture systems such as Vicon and Qualisys, or high-speed video capture) that allows for criterion validation. Therefore, this paper presented concurrent validity alongside ICC reliability and absolute agreement to allow users of the Trackman 4 and Mevo ​+ ​units to be confident in the understanding and interpretation of data they collect.

A single participant design was used in this study. This approach has been used elsewhere [8] and was taken so that data collected focused on consistency and agreement between the launch monitors investigated. Additionally, between-participant variability has also been investigated elsewhere [3]. The single participant demonstrated substantial variability in their performance to comprise “normal” conditions (see tables and range values) although it is possible that the single participant design may introduce less variability than a between-participant design as the participant will have a level of idiosyncrasy in their swing characteristics and club delivery profile. It was outside the scope of this study to investigate how launch monitors perform in extreme conditions such as with exceptionally slow or fast swings and therefore the consistency and agreement data presented herein cannot be extrapolated with these cohorts. However, these applications will be limited and do not represent “typical” usage from club fitters, coaches, or golfers themselves.

Lastly, when conducting consistency and agreement investigations using multiple systems concurrently, the manual alignment of the two launch monitors to the same target line is a potential source of error [13]. The approach taken here of calibrating Trackman 4 first, and then aligning Mevo ​+ ​to the Trackman 4 target line allowed for alignments to be matched, but the authors note that this is constrained by the pixel resolution rate on a computer tablet. Although marginal, a very small misalignment of one degree would create a four-yard dispersion differential over a shot of 250 yards carry and have similar impacts on directional metrics, such as dispersion and club path. Practitioners and researchers are therefore encouraged to ensure that manual alignment is conducted on digital devices with large screens and high pixel resolution to ensure best possible accuracy.

## 5 Conclusion

This study demonstrates the Mevo ​+ ​system provides moderate to excellent consistency with Trackman 4 across all variables during indoor golf swings, with the exception of angle of attack with pitching wedge and 7-iron. Mevo ​+ ​provides differences in absolute values across variables, particularly for angle of attack, dynamic loft, club speed, and spin rate, it does however, maintain consistency across these variables. Users may find a level of interchangeability between devices where consistency and absolute agreement are high but should consider this when comparing performance across launch monitors or when using Mevo ​+ ​for club fitting, performance enhancement, or research purposes.

Lastly, we provide the following practical recommendations.

• To improve comparability between systems, only shots where all fields of data were captured should be used in applied practice.
• Mevo ​+ ​users should be rigorous with visual inspection of data collected, particularly for unrealistically high “measured” spin rates as occurred here with a very small number of shots.
• Most Mevo ​+ ​data demonstrated consistency with Trackman 4. However, absolute agreement was not always of comparable strength, suggesting that overall, Mevo ​+ ​demonstrates consistency but does provide different absolute values in a systematic, repeatable manner.
• Angle of attack, and dynamic loft were two variables that demonstrated poor consistency and absolute agreement and therefore should not be compared to Trackman 4 data.
• When calibrating Mevo+, a large screen with high resolution should be used. This allows accuracy to be increased as small screens offer less ability to match a target line. A degree of difference in calibration alignment will lead to a four-yard dispersion (side) difference over 250 yards of carry and slightly different values for launch direction etc. This would not be the fault of the system, but rather the user.

## Funding

None.

## Declaration of competing interest

The authors declare that they have no known competing financial interests or personal relationships that could have appeared to influence the work reported in this paper.
